# Evaluating interaction forces between BSA and rabbit anti-BSA in sulphathiazole sodium, tylosin and levofloxacin solution by AFM

**DOI:** 10.1186/1556-276X-6-579

**Published:** 2011-11-03

**Authors:** Congzhou Wang, Jianhua Wang, Linhong Deng

**Affiliations:** 1 Key Laboratory of Biorheological Science and Technology, Ministry of Education, Chongqing University, 400044 Chongqing, China; 2 Institute of Biochemistry and Biophysics, College of Bioengineering, Chongqing University, 400044 Chongqing, China

## Abstract

Protein-protein interactions play crucial roles in numerous biological processes. However, it is still challenging to evaluate the protein-protein interactions, such as antigen and antibody, in the presence of drug molecules in physiological liquid. In this study, the interaction between bovine serum albumin (BSA) and rabbit anti-BSA was investigated using atomic force microscopy (AFM) in the presence of various antimicrobial drugs (sulphathiazole sodium, tylosin and levofloxacin) under physiological condition. The results show that increasing the concentration of tylosin decreased the single-molecule-specific force between BSA and rabbit anti-BSA. As for sulphathiazole sodium, it dramatically decreased the specific force at a certain critical concentration, but increased the nonspecific force as its concentration increasing. In addition, the presence of levofloxacin did not greatly influence either the specific or nonspecific force. Collectively, these results suggest that these three drugs may adopt different mechanisms to affect the interaction force between BSA and rabbit anti-BSA. These findings may enhance our understanding of antigen/antibody binding processes in the presence of drug molecules, and hence indicate that AFM could be helpful in the design and screening of drugs-modulating protein-protein interaction processes.

## 1. Introduction

A molecular level understanding of protein-protein interactions is fundamentally important in the life sciences. A number of human diseases are closely related to the protein-protein association or dissociation events and thus probing and characterizing these interactions have become increasingly significant in the development of novel drugs and medical diagnostics [1-4]. Different solution conditions, such as pH, temperature, ion species, and strength, may influence the protein-protein interactions as previous studies have demonstrated [5-7]. This is particularly important in drug discovery and the computer-aided drug design (CADD) method has identified molecules modifying protein-protein interactions as potential drug candidates [8,9]. However, the computer studies do not provide more detailed information on forces at nanoscale-to-molecular scale that influence protein-protein interactions, which would allow us to better understanding the factors of drug molecules affecting the interactions. Therefore, it is still challenging to evaluate the protein-protein interactions, such as that between antigen and antibody, in the presence of drug molecules in physiological liquid.

**Figure 1:**
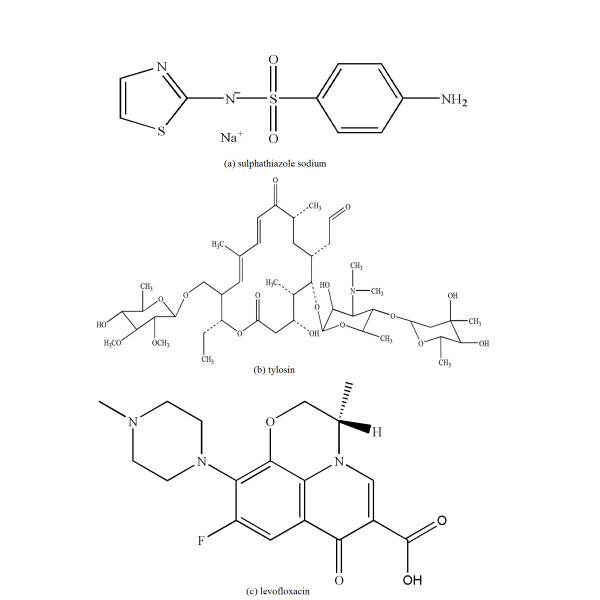
**Chemical structures of drug molecules**. **(a) **Chemical structure of sulphathiazole sodium. **(b) **Chemical structure of tylosin. **(c) **Chemical structure of levofloxacin.

Bovine serum albumin (BSA) is the major protein constituent of blood plasma and it facilitates the disposition and transport of various exogenous and endogenous ligands to the specific targets. Many drugs and other bioactive small molecules bind reversibly to BSA [10,11]. Consequently, it is important to study the drugs effect on this protein. Sulphathiazole sodium, tylosin, and levofloxacin are antimicrobial drugs that belong to sulphonamides, macrolides, and fluoroquinolone family, respectively. (The chemical structures of these three drugs are shown in Figure 1.) The distribution, antimicrobial activity, and toxicity of these drugs are strongly dependent on the extent of their binding by serum albumin. There have been several spectroscopic studies on fluorescence quenching and structure analysis of serum albumin induced by these drugs or other bioactive small molecules [12-14]. Nevertheless, no investigations have been made of the mechanical behavior of BSA in the presence of these drugs.

By using an atomic force microscopy (AFM), it has been possible to measure directly the specific and nonspecific force between proteins at molecular scale. AFM is widely applied to characterize biological molecular recognition processes because of its high force sensitivity and the capability of operating under different physiological conditions [15-18]. We have previously testified an experimental method for the characterization of the specific and nonspecific interaction force between human immunoglobulin G (IgG) and rat anti-human IgG in phosphate buffered saline (PBS). Self-assembled monolayer (SAM) method was used for sample preparation and AFM was employed for interaction force measurement [19]. SAM method has been proved to be a facile and effective way to form well-defined and controlled films for AFM sample preparation [20,21]. In this article, we investigated the interaction between BSA and rabbit anti-BSA when it was measured by AFM in either PBS or PBS solution containing one of the three antimicrobial drugs (sulphathiazole sodium, tylosin, and levofloxacin) under physiological conditions. The results suggest that these three drugs may adopt different mechanisms to affect the interaction force between BSA and rat-anti BSA.

## 2. Experimental methods and materials

To investigate protein-protein interactions through AFM, we used a thiol-based SAM for protein immobilization because of its effectiveness and simplicity, which is similar to our previous report [22]. In brief, sulphur-containing molecules (thiols, sulphides, and disulphides) have a strong affinity for gold and will interact with it in near covalent manner. Therefore, when gold is immersed into a solution of thiols such as 16-mercaptohexadecanoic acid (MHA), the thiol molecules will spontaneously react with gold and form a SAM of thiols on the gold surface with tightly packed and well-ordered chains. The terminal end of the thiol-based SAM consists of carboxyl tail groups that can be activated by the 1-ethyl-3-(dimethylaminopropyl) carbodi-imide hydrochloride (EDC) and N-hydroxysulphosuccinimide (NHS). The activated SAM can then be soaked into protein solution to form protein layer.

### 2.1. Gold-coated substrate

Gold-coated substrates were prepared by vapor deposition of gold onto freshly cleaved mica in a high vacuum evaporator at approx. 10^-7 ^Torr. Mica substrates were preheated to 325°C for 2 h by a radiator heater before deposition. Evaporation rates were 0.1-0.3 nm/s, and the final thickness of the gold films was approx. 200 nm. A chromium layer was also vapor deposited and sandwiched between the gold and mica to strengthen the adhesion between the surfaces. The gold-coated substrate was then annealed in H_2 _flame for 1 min before use.

### 2.2. SAM of thiols on gold surface

The bare gold-coated substrate prepared as above was thoroughly cleaned in hot piranha solution (*v*/*v *H_2_SO_4_:H_2_O_2 _= 3:1) for 30 min. The gold-coated substrate was then immersed into the ethanol solution of 1 mM MHA for 24 h to produce the thiol-based SAM on the gold surface, and unbound thiols were removed by ultrasonication in pure ethanol for 2 min. The prepared SAM was then rinsed sequentially with pure ethanol, ultra pure water, and finally dried in a N_2 _stream before use.

### 2.3. Protein immobilization onto the SAM

BSA was covalently immobilized on a gold-coated substrate through the condensation reaction between the amino groups in the protein and the carboxyl groups on the gold-coated substrate [23]. In brief, SAM with carboxylic acid terminal groups was activated by 2 mg/mL NHS and 2 mg/mL EDC in PBS for 1 h, and subsequently rinsed thoroughly with ultra pure water, and dried in N_2 _stream. The activated SAM was then immersed into 5 μg/mL BSA in PBS at 4°C for 12 h. Finally, the prepared sample of protein layer was kept in PBS at 4°C until use.

### 2.4. Functionalization of AFM tip

Functionalized AFM tip with rabbit anti-BSA coating was prepared similarly as described above.

### 2.5. Measurement of antigen-antibody adhesion force by AFM in drug solutions

Adhesion force between BSA and rabbit anti-BSA was measured by AFM using Benyuan CSPM 5000 scanning probe microscope (Benyuan Co., China). The functionalized AFM tip scanned across the well-ordered protein monolayer. At a given location, the tip was moved toward the surface of the monolayer and retracted. When the tip approached the monolayer surface it would deflect because of the antigen-antibody interaction force, which would be detected as a "voltage-displacement" signal and converted into a "force-displacement" curve [24,25]. Because the tip was considered an elastic cantilever, its deflection was determined by the force (*F*) exerted on it following Hooke's law, i.e., *F *= *k *× *d*, where *d *is the deflection, *k *is the spring constant of the cantilever tip. In general, *k *should be small for AFM to minimize measurement noise. In this study, commercially available Si_3_N_4 _cantilever tip (BudgetSensors^®^, Innovative Solutions Bulgaria Ltd., Bulgaria) was used of which the spring constant, calibrated by thermal fluctuation method [26], was 0.2-0.3 N/m. The tip has a pyramidal geometry. Its tip radius is about 25 nm and the thickness of the gold layer is 70 nm.

All force measurements were performed using contact mode AFM at room temperature (25°C). The functionalized AFM tip with rabbit anti-BSA was used to measure the adhesion force between the substrate of BSA and the tip of rabbit anti-BSA in PBS as control experiment. The retraction velocity was estimated to be 0.04 μm/s, and all the measurements were observed under this condition. From the "force-displacement" curve, the adhesion force was calculated. Measurement was repeated about 50-55 times at each of 5 randomly selected locations across the protein monolayer on the gold substrate. To mimic the various antimicrobial drug solution media, the PBS in control experiment was separately changed to sulphathiazole sodium, tylosin, and levofloxacin solution (one of the drugs dissolved in PBS) over a concentration range of 10-70 mM. A complete series of measurements in the control and in each of the drug solutions were conducted using the same functionalized AFM tip. The five selected locations across the protein monolayer in control experiment were measured in the drug solutions.

### 2.6. AFM imaging

All images were acquired using Benyuan CSPM 5000 scanning probe microscope (Benyuan Co., China) equipped with a 1.6-μm E scanner. Commercial Si_3_N_4 _cantilevers (BudgetSensors) with resonant frequency of 200 kHz were used. AFM worked with tapping mode in PBS and drug solutions at typical scanning rate of 2.0 Hz and scanning size of 1000 nm × 1000 nm. The roughness of surfaces in different solutions was analyzed by CSPM Image 4.62 software program (provided by the manufacturer).

### 2.7. Materials

16-MHA, 1-ethyl-3-(dimethylaminopropyl) carbodi-imide hydrochloride (EDC), NHS, sulphathiazole sodium, tylosin, and levofloxacin were purchased from Sigma Aldrich Chemical Co. and used as-received. PBS (140 mM NaCl, 3 mM KCl, pH 7.4) and ethanol (guaranteed grade) were purchased from Merck Co., and ultra pure water (resistivity of 18.2 MΩ cm) was obtained by Millpore purification system. BSA and rabbit anti-BSA were purchased from Biosun Co. (China).

## 3. Results and discussion

**Figure 2:**
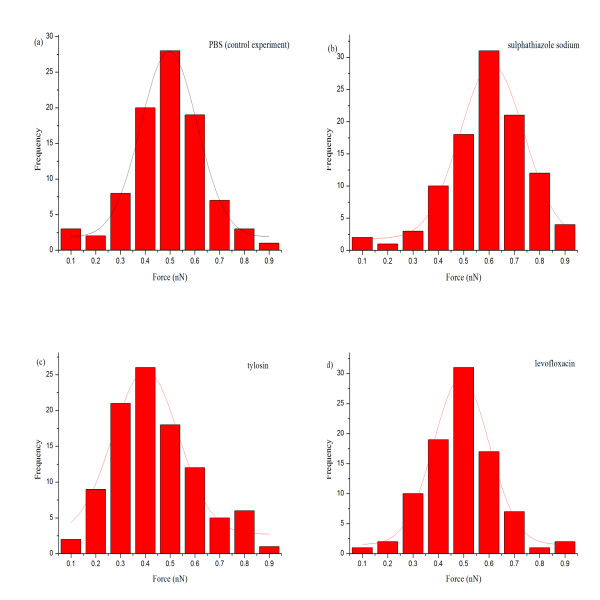
**Distribution histograms of all measured adhesion forces in different kinds of physiological liquid**. **(a) **Distribution histograms of measured adhesion forces in PBS. **(b) **Distribution histograms of measured adhesion forces in sulphathiazole sodium solution (10 mM). **(c) **Distribution histograms of measured adhesion forces in tylosin solution (10 mM). **(d) **Distribution histograms of measured adhesion forces in levofloxacin solution (10 mM). The distributions of the adhesion forces could be fitted to Gaussian models.

Our previous research justified SAM for protein immobilization and AFM for interaction force measurement [19]. The same combined method was adopted for BSA and rabbit anti-BSA system because it is relatively simple, sensitive and reliable. The adhesion forces between BSA and rabbit anti-BSA in PBS (control experiment) and their probability distribution were calculated from repeated measurements and plotted in Figure 2a. The distribution of the adhesion forces in PBS could be fitted with Gaussian models and varied between 0.1 and 0.9 nN. The majority of them were between 0.3 and 0.7 nN. Considering the adhesion force measured by AFM was not that of a single antigen-antibody pair, but rather a collective result of interaction forces from multiple antigen/antibody pairs, the Poisson statistical method developed by Beebe et al. [27,28] could be used to determine the unbinding force required to separate a single pair of antigen and antibody molecules. The advantage of this method was verified that it provided an accurate calculation of single-molecule specific force in the presence of moderate-to-large variation or noise of various types [29]. As defined by the Poisson distribution, the mean value equals the variance of the number (*n*) of interacting antigen-antibody pairs. Provided that the measured total interaction force is composed of a finite number of discrete interacting antigen-antibody pairs within a fixed contact area, the specific force between a single antigen-antibody pair (*F_i_*) and possible nonspecific interaction force (*F*_0_) can be derived from the slope and interception of the linear regression curve of the variance ($\sigma_{m}^{2}$) versus the mean (*μ_m_*) of the measured total adhesion force as $\sigma_{m}^{2}=\mu_{m}F_{i}-F_{i}F_{0}$[27].

**Figure 3:**
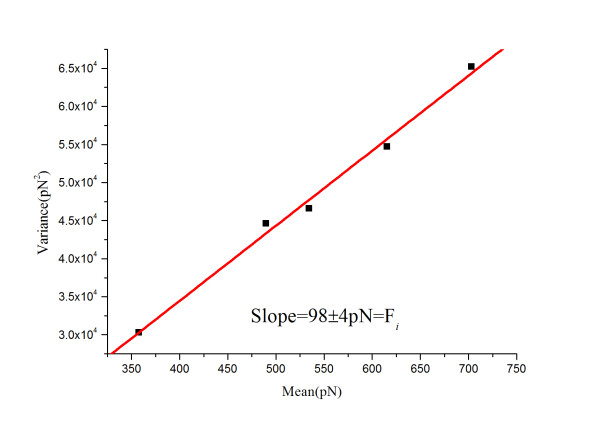
**The variance ($\sigma_{m}^{2}$) was plotted versus the mean (μm) of the measured interaction forces between BSA and rabbit anti-BSA in PBS**. Each data point represents a dataset taken at one of the five different sample locations. Details of the datasets are given in Table 1 (*R *= 0.9902).

The total adhesion forces between BSA and rabbit anti-BSA were measured repeated for 50-55 times at each of several randomly chosen locations of the BSA monolayer in PBS, and the mean (*μ_m_*) and variance ($\sigma_{m}^{2}$) of these measurements are given in Table 1, and plotted with linear regression as shown in Figure 3. From these results, the specific force between a single pair of BSA and rabbit anti-BSA, *F_i _*and the nonspecific force, *F*_0_, were calculated as 98 ± 4 and 48 pN, respectively. This level of specific adhesion force was well within the range of 35-165 pN that has been reported as the estimated range of force required to rupture a single antigen-antibody complex [30]. The successful measurement of BSA and rabbit anti-BSA adhesion interactions in PBS (control experiment) demonstrates that both proteins retained their folded conformation and remained functional following our immobilization protocol.

**Table 1:** Adhesion forces between BSA and rabbit anti-BSA measured at five different locations on BSA substrate in PBS, sulphathiazole sodium, tylosin and levofloxacin solution (10 mM)

Solution medium	Location	Mean force *μ_m _*(pN)	Variance of force *σ_m_*^2 ^(×10^4 ^pN^2^)	Number of measurement (*n*)
PBS (control experiment)	1	357.6	3.03	52
	2	489.7	4.46	50
	3	534.4	4.66	53
	4	615.4	5.47	53
	5	703.1	6.52	52
PBS+ sulphathiazole sodium	1	391.4	3.15	52
	2	542.1	4.50	50
	3	593.3	5.12	52
	4	673.1	6.18	53
	5	779.5	7.01	53
PBS+ tylosin	1	304.7	1.35	50
	2	426.2	2.12	53
	3	489.5	2.27	53
	4	565.4	2.73	52
	5	667.8	3.35	52
PBS+ levofloxacin	1	369.1	3.23	52
	2	491.9	4.36	50
	3	541.6	5.15	53
	4	621.1	5.79	53
	5	705.2	6.60	52

**Figure 4:**
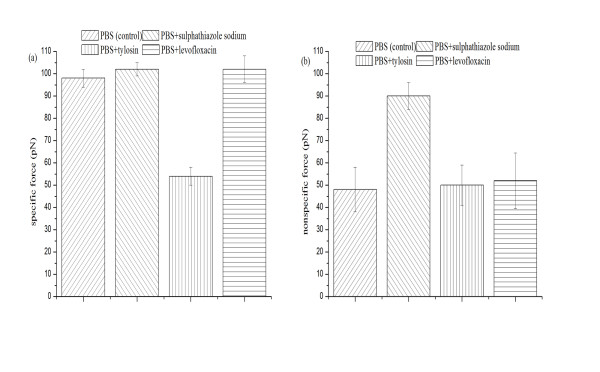
**Bar plot summarizing the specific force between a single pair of BSA and rabbit anti-BSA and the nonspecific force in PBS (as a reference), sulphathiazole sodium, tylosin and levofloxacin solution (10 mM)**. **(a) **The specific force between a single pair of BSA and rabbit anti-BSA, *F_i_*. **(b) **The nonspecific force between BSA and rabbit anti-BSA, *F_0_*.

Figure 2b-d shows the representative histograms of adhesion forces of BSA and rabbit anti-BSA in sulphathiazole sodium, tylosin, and levofloxacin solution (10 mM), respectively. The mean (*μ_m_*) and variance ($\sigma_{m}^{2}$) of these measurements are given in Table 1, and then plotted with linear regression. The specific force between a single pair of BSA and rabbit anti-BSA, *F_i _*and the nonspecific force, *F*_0 _in PBS and the three drug solutions are summarized in Figure 4. It is observed that the specific force in tylosin solution is smallest in all solutions (Figure 4a). This was expected because the spatial structure of tylosin molecule is biggest of these three drug molecules, and when tylosin molecules absorb on surfaces of BSA and rabbit anti-BSA, they may cover available binding sites and weaken the specific adhesion force between BSA and rabbit anti-BSA. According to the definition of the Poisson distribution method, the chemical and hydrogen bonds are considered as specific interactions, whereas the electrostatic interactions are counted toward part of nonspecific interactions [31]. Tylosin molecules may hinder the formation of chemical and hydrogen bonds between BSA and rabbit anti-BSA. This result is in line with our previous reports that binding was inhibited when surface epitopes were blocked by excess antibody applied before AFM was performed [19,32]. Kim et al. [33] found polymyxin B affected the molecular interaction between lipopolysaccharide (LPS) binding protein-LPS complex and the receptor protein using AFM and different structures of the drugs resulted in different bonding forces. Kanapathipillai et al. [34] depicted that the behavior of solute was highly dependent on its structure and some molecules could play a key role in the prion inhibition mechanism because they could interfere with the hydrogen bonded monomer-monomer interactions of prion proteins.

The largest nonspecific force observed in sulphathiazole sodium solution (Figure 4b) could be attributed to the effect of increasing solution ionic strength (IS). Both BSA and rabbit anti-BSA are negatively charged when immersed in solution (pH 7.4), as the isoelectric points of BSA and rabbit anti-BSA are 4.7, 4.8-5.2, respectively [35]. Increasing the solution IS compressed the thickness of the electrostatic double layer surrounding proteins, and finally resulted in an increase in nonspecific adhesion. This phenomenon is qualitatively consistent with predictions based on DLVO (Derjaguin, Landau, Verwey, Overbeek) theory as an increase in the solution IS will reduce the range of electrostatic repulsion between two negatively charged surfaces [36,37]. Similar effect was reported by Javid et al. [6]. They observed that the positive charge on the lysozyme molecule was screened by the salt anions as the salt concentration increased, hence diminishing the strong repulsive protein-protein interactions. In addition, increasing the solution IS may disrupt the hydration shell coating on protein surfaces and thus reduce repulsive interactions between the two interacting surfaces [38]. Benítez et al. [39] studied the effect of IS on the stability of apple juice particles which are mainly composed of proteins and carbohydrates. They concluded that increasing IS resulted in reduction of surface charge and hydration constant, and led to an increase in adhesion. Compared with tylosin molecule, the spatial structure of sulphathiazole sodium salt in solution is smaller and sulphathiazole sodium molecules may not cover available binding sites and weaken the specific adhesion between antigen and antibody. In levofloxacin solution, the specific adhesion force and nonspecific force are almost equal to the force values in PBS. This suggests that levofloxacin as a small nonionic drug may not affect the interactions of BSA and rabbit anti-BSA because of neither bigger spatial structure of levofloxacin molecule nor increasing IS in solution.

**Figure 5:**
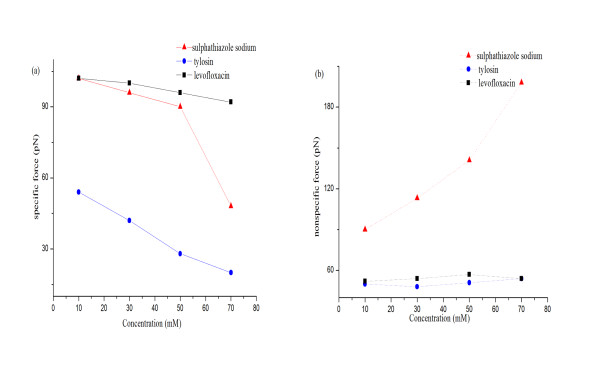
**The specific and nonspecific forces between BSA and rabbit anti-BSA with changing concentrations of the three drug solutions**. **(a) **The specific force between a single pair of BSA and rabbit anti-BSA, *F_i_*. **(b) **The nonspecific force between BSA and rabbit anti-BSA, *F_0_*.

**Figure 6:**
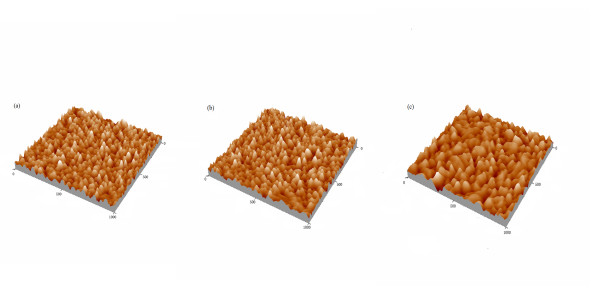
**Topographies of the BSA monolayer in different kinds of physiological liquid recorded by tapping mode AFM**. **(a) **Topography of the BSA monolayer in PBS. **(b) **Topography of the BSA monolayer in 50 mM sulphathiazole sodium solution. **(c) **Topography of the BSA monolayer in 70 mM sulphathiazole sodium solution. The scanning size is 1000 nm × 1000 nm.

According to initial forces data, the drugs concentration effect on the specific and nonspecific forces was further obtained (Figure 5). The specific forces in tylosin solution decreased for the range of drug concentration examined here. As the increase of drug concentration, we may conclude that tylosin molecule reduced the specific force between BSA and rabbit anti-BSA by covering available binding sites because of its bigger spatial structure. In sulphathiazole sodium solution, a critical concentration of 70 mM sulphathiazole sodium was identified. At this concentration, the specific force dramatically decreased from 90 to 48 pN. This phenomenon is in contrast to what we would expect that the presence of sulphathiazole sodium did not affect the specific force of BSA and rabbit anti-BSA. We believe that this reduction in specific force is a result of the change in the initial conformation of the BSA monolayer in a solution at a critical solution IS. In the low IS solution, the BSA monolayer would be in a more unfolded state and further expanded into solution, providing more potential binding sites when antibody was pressed onto the antigen monolayer. However, as the solution IS was increased to a critical value, the monolayer would become more folded and compressed, forming a denser core and providing fewer specific interaction sites. The more condensed structure of the antigen monolayer at the higher solution IS could result in the formation of weaker bonds with antibody, leading to a smaller specific force as observed here [40]. This speculation is supported by the observed changes in BSA monolayer conformation shown in Figure 6. The surface change is quantitatively indicated by surface roughness. For BSA monolayer in PBS (Figure 6a) and 50 mM sulphathiazole sodium solution (Figure 6b), the roughness (value of root mean square) was calculated to be 1.59 and 1.57 nm, respectively. For BSA monolayer in 70 mM sulphathiazole sodium solution (Figure 6c), the roughness was only 0.95 nm. No conformational changes occurred in BSA monolayer in the presence of tylosin and levofloxacin (data not shown). This suggests that the monolayer will become more folded and compressed at the critical solution IS of sulphathiazole sodium. This observation is similar to the finding of Lazar et al. [41]. In their study, it was shown that BSA formed films with different micro-structures in the presence of various sodium salts. The concentration of sulphathiazole sodium affected the nonspecific force, as shown by a higher nonspecific force with an increase in the concentration of sulphathiazole sodium. We believe that increasing the concentration of sulphathiazole sodium compressed the thickness of the electrostatic double layer surrounding proteins and disrupted the hydration shell coating on protein surfaces, so it eventually resulted in an increase in nonspecific adhesion. The variation of levofloxacin concentration did not clearly influence the specific and nonspecific force of BSA and rabbit anti-BSA. This indicates that the presence of levofloxacin as a small nonionic drug did not affect significantly the interactions of BSA and rabbit anti-BSA in the solution.

## 4. Conclusions

The interaction between BSA and rabbit anti-BSA was investigated by AFM in PBS and three antimicrobial drug (sulphathiazole sodium, tylosin and levofloxacin) solutions under physiological conditions. The results suggest that increasing the concentration of tylosin solution decreased the single-molecule-specific force, demonstrating the important contribution of tylosin molecules spatially covering available binding sites to decreased specific adhesion force. At a certain critical concentration of sulphathiazole sodium, the single-molecule-specific force decreased dramatically because of the change in the initial conformation of the BSA monolayer. The nonspecific force increased as the concentration of sulphathiazole sodium increased, suggesting that sulphathiazole sodium as an ionic drug increasing solution IS was the dominant mechanism of nonspecific force. The presence of levofloxacin as a small nonionic drug did not significantly affect the interactions of BSA and rabbit anti-BSA in the solution. These findings may enhance our understanding of antigen/antibody binding processes in the presence of drug molecules, and hence indicate the AFM could be helpful in the design and screening of drugs modulating protein-protein interaction processes.

## Competing interests

The authors declare that they have no competing interests.

## Authors' contributions

CW carried out the AFM measurement and data analysis. JW conceived of the study, and participated in its design and coordination. LD participated in the revising the manuscript. All authors read and approved the final manuscript.

## References

[B1] Calarese DA Scanlan CN Zwick MB Deechongkit S Mimura Y Kunert R Zhu P Wormald MR Stanfield RL Roux KH Kelly JW Rudd PM Dwek RA Katinger H Burton DR Wilson IA Antibody domain exchange is an immunological solution to carbohydrate cluster recognition Science 2003 300 2065 2071 10.1126/science.1083182 12829775

[B2] Carroll MC The complement system in regulation of adaptive immunity Nat Immunol 2004 5 981 986 10.1038/ni1113 15454921

[B3] Peretz D Williamson RA Kaneko K Vergara J Leclerc E Schmitt-Ulms G Mehlhorn IR Legname G Wormald MR Rudd PM Dwek RA Burton DR Prusiner SB Antibodies inhibit prion propagation and clear cell cultures of prion infectivity Nature 2001 412 739 743 10.1038/35089090 11507642

[B4] Li H Sethuraman N Stadheim TA Zha D Prinz B Ballew N Bobrowicz P Choi BK Cook WJ Cukan M Houston-Cummings NR Davidson R Gong B Hamilton SR Hoopes JP Jiang Y Kim N Mansfield R Nett JH Rios S Strawbridge R Wildt S Gerngross TU Optimization of humanized IgGs in glycoengineered Pichia pastoris Nat Biotechnol 2006 24 210 215 10.1038/nbt1178 16429149

[B5] Yu J Warnke J Lyubchenko YL Nanoprobing of α-synuclein misfolding and aggregation with atomic force microscopy Nanomedicine 2011 7 146 152 10.1016/j.nano.2010.08.001 20817126

[B6] Javid N Vogtt K Krywka C Tolan M Winter R Protein-protein interactions in complex cosolvent solutions Chemphyschem 2007 8 679 689 10.1002/cphc.200600631 17328089

[B7] Jones OG Adamcik J Handschin S Bolisetty S Mezzenga R Fibrillation of β-lactoglobulin at low pH in the presence of a complexing anionic polysaccharide Langmuir 2010 26 449 458 10.1021/la1026619 20968310

[B8] Veselovsky AV Archakov AI Inhibitors of protein-protein interactions as potential drugs Curr Comput Aid Drug 2007 3 51 58

[B9] Arkin MR Wells JA Small-molecule inhibitors of protein-protein interactions: progressing towards the dream Nat Rev Drug Discov 2004 3 301 317 10.1038/nrd1343 15060526

[B10] Kumar CV Buranaprapuk A Opiteck GJ Moyer MB Jockusch S Turro NJ Photochemical protease: site-specific photocleavage of hen egg lysozyme and bovine serum albumin Proc Natl Acad Sci 1998 95 10361 10366 10.1073/pnas.95.18.10361 PMC27899 9724708

[B11] Olson RE Christ DD Plasma protein binding of drugs Ann Rep Med Chem 1996 31 327 336

[B12] Wen MG Zhang XB Tian JN Ni SH Bian HD Huang YL Liang H Binding interaction of xanthoxylin with bovine serum albumin J Solution Chem 2009 38 391 401

[B13] Hu YJ Liu Y Xiao XH Investigation of the interaction between berberine and human serum albumin Biomacromolecules 2009 10 517 521 10.1021/bm801120k 19173654

[B14] Zhang G Keita B Craescu CT Miron S Oliveira P Nadjo L Molecular interactions between Wells-Dawson type polyoxometalates and human serum albumin Biomacromolecules 2008 9 812 817 10.1021/bm701120j 18266320

[B15] Allison DP Hinterdorfer P Han W Biomolecular force measurements and the atomic force microscope Curr Opin Biotechnol 2002 13 47 51 10.1016/s0958-1669(02)00283-5 11849957

[B16] Lee CK Wang YM Huang LS Lin S Atomic force microscopy: determination of unbinding force, off rate and energy barrier for protein-ligand interaction Micron 2007 38 446 461 10.1016/j.micron.2006.06.014 17015017

[B17] Muller DJ Dufrene YF Atomic force microscopy as a multifunctional molecular toolbox in nanobiotechnology Nat Nanotechnol 2008 3 261 269 10.1038/nnano.2008.100 18654521

[B18] Okada T Sano M Yamamoto Y Muramatsu H Evaluation of interaction forces between profilin and designed peptide probes by atomic force microscopy Langmuir 2008 24 4050 4055 10.1021/la703344u 18335966

[B19] Lv ZJ Wang JH Deng LH Chen GP Probing specific interaction forces between human IgG and rat anti-human igg by self-assembled monolayer and atomic force microscopy Nanoscale Res Lett 2010 5 1032 1038 10.1007/s11671-010-9598-x PMC2893755 20671785

[B20] Ferretti S Paynter S Russell DA Sapsford KE Richardson DJ Self-assembled monolayers: a versatile tool for the formulation of bio-surfaces Trends Analyt Chem 2000 19 530 540

[B21] Love JC Estroff LA Kriebel JK Nuzzo RG Whitesides GM Self-assembled monolayers of thiolates on metals as a form of nanotechnology Chem Rev 2005 105 1103 1160 10.1021/cr0300789 15826011

[B22] Lv ZJ Wang JH Deng LH Chen GP Preparation and characterization of covalently binding of rat anti-human IgG monolayer on thiol-modified gold surface Nanoscale Res Lett 2009 4 1403 1408 10.1007/s11671-009-9412-9 PMC2893859 20652126

[B23] Wakayama J Sekiguchi H Akanuma S Ohtani T Sugiyama S Methods for reducing nonspecific interaction in antibody-antigen assay via atomic force microscopy Anal Biochem 2008 380 51 58 10.1016/j.ab.2008.05.036 18559251

[B24] Hinterdorfer P Dufrene YF Detection and localization of single molecular recognition events using atomic force microscopy Nat Methods 2006 3 347 355 10.1038/nmeth871 16628204

[B25] Briand E Gu C Boujday S Salmain M Herry JM Pradier CM Functionalisation of gold surfaces with thiolate SAMs: ^t^opography/bioactivity relationship-a combined FT-RAIRS, AFM and QCM investigation Surf Sci 2007 601 3850 3855

[B26] Hutter JL Bechhoefer J Calibration of atomic force microscope tips Rev Sci Instrum 1993 64 1868 1873

[B27] Lo YS Huefner ND Chan WS Stevens F Harris JM Beebe TP Specific interactions between biotin and avidin studied by atomic force microscopy using the Poisson statistical analysis method Langmuir 1999 15 1373 1382

[B28] Liu W Parpura V Single molecule probing of SNARE proteins by atomic force microscopy Ann N Y Acad Sci 2009 1152 113 120 10.1111/j.1749-6632.2008.03991.x PMC2702522 19161382

[B29] Jiang YX Qin F Ma XY Li YQ Bai CL Fang XH Measuring specific interaction of transcription factor ZmDREB1A with its DNA responsive element at the molecular level Nucleic Acids Res 2004 32 e101 10.1093/nar/gnh100 PMC484199 15249597

[B30] Dammer U Hegner M Anselmetti D Wagner P Dreier M Huber W Guntherodt HJ Specific antigen/antibody interactions measured by force microscopy Biophys J 1996 70 2437 2441 10.1016/S0006-3495(96)79814-4 PMC1225221 9172770

[B31] Abu-Lail NI Camesano TA Specific and nonspecific interaction forces between escherichia coli and silicon nitride determined by poisson statistical analysis Langmuir 2006 22 7296 7301 10.1021/la0533415 16893229

[B32] Lv ZJ Wang JH Deng LH Chen GP Imaging recognition events between human IgG and rat anti-human IgG by atomic force microscopy Int J Biol Macromol 2010 47 661 667 10.1016/j.ijbiomac.2010.08.017 20813125

[B33] Kim SJ Jang S Kim U Cho K AFM studies of inhibition effect in binding of antimicrobial peptide and immune proteins Langmuir 2007 23 10438 10440 10.1021/la702173e 17854214

[B34] Kanapathipillai M Ku SH Girigoswami K Park CB Small stress molecules inhibit aggregation and neurotoxicity of prion peptide 106-126 Biochem Bioph Res Commun 2008 365 808 813 10.1016/j.bbrc.2007.11.074 18039468

[B35] Ge S Kojio K Takahara A Kajiyama T Bovine serum albumin adsorption onto immobilized organotrichlorosilane surface: ^i^nfluence of the phase separation on protein adsorption patterns J Biomater Sci Polym Ed 1998 9 131 150 10.1163/156856298x00479 9493841

[B36] Leckband D Sivasankar S Forces controlling protein interactions: theory and experiment Colloids Surf B Biointerface 1999 14 83 97

[B37] Oliveira R Understanding adhesion: a means for preventing fouling Exp Therm Fluid Sci 1997 14 316 322

[B38] Besseling NAM Theory of hydration forces between surfaces Langmuir 1997 13 2113 2122

[B39] Benítez EI Genovese DB Lozano JE Effect of pH and ionic strength on apple juice turbidity: application of the extended DLVO theory Food Hydrocolloid 2007 21 100 109

[B40] Xu LC Vadillo-Rodriguez V Logan BE Residence time, loading force, pH, and ionic strength affect adhesion forces between colloids and biopolymer-coated surfaces Langmuir 2005 21 7491 7500 10.1021/la0509091 16042484

[B41] Lazar AN Shahgaldian P Coleman AW Anion recognition effects in the structuring of bovine serum albumin films J Supramol Chem 2001 1 193 199

